# Caregivers’ experiences of a home support program after the hospital discharge of an older family member: a qualitative analysis

**DOI:** 10.1186/s12913-019-4042-0

**Published:** 2019-04-11

**Authors:** Susan Slatyer, Samar M. Aoun, Keith D. Hill, Debbie Walsh, Dee Whitty, Christine Toye

**Affiliations:** 1 Discipline of Nursing, College of Science, Health, Engineering and Education, Murdoch University, Murdoch, Western Australia 6150 Australia; 2 School of Nursing, Midwifery and Paramedicine, Curtin University , GPO Box U1987, Perth, Western Australia 6845 Australia; 3 Centre for Nursing Research, Sir Charles Gairdner Hospital , Hospital Avenue, Nedlands, Western Australia 6009 Australia; 4 School of Psychology & Public Health, La Trobe University, Boondoora, 3086 Victoria Australia; 5 Perron Institute for Neurological and Translational Science, Verdun Street, Nedlands, Western Australia 6009 Australia; 6 Faculty of Health Sciences, Curtin University , GPO Box U1987, Perth, 6845 Western Australia Australia; 7 Medical Division, Sir Charles Gairdner Hospital, Hospital Avenue, Nedlands, Western Australia 6009 Australia

**Keywords:** Caregiver needs, Older people, Qualitative research, Nursing, Post-discharge

## Abstract

**Background:**

The ageing global population has seen increasing numbers of older people living with chronic health problems, declining function, and frailty. As older people seek to live out their years at home, family members, friends and neighbours (informal caregivers) are increasingly relied upon for support. Moreover, pressured health systems and shorter hospital length of stay mean that informal caregivers can find themselves supporting the older person who is still unwell after discharge. The Further Enabling Care at Home (FECH) program was developed as a nursing outreach intervention designed to systematically address support needs of family caregivers of older people after hospital discharge to sustain their home-based caregiving. The objective of this study was to explore the experiences of informal caregivers who participated in the FECH program after an older family member’s discharge from hospital.

**Methods:**

The study employed a qualitative descriptive design. Caregivers of older people discharged home from a Medical Assessment Unit in an Australian hospital who were included in the program were interviewed to explore their experiences and perceptions of the FECH program. Data were audio-recorded, transcribed, and subjected to thematic analysis.

**Results:**

Twenty-one family caregivers (81% female, aged 25–89 years) participated in the interviews. Themes emerging were ‘The experience of caregiving’; ‘The experience of receiving FECH program support’; and ‘Caregivers’ suggestions for improvement’. Caregivers indicated that reflective discussions with the FECH nurse enabled them to recognise the complexity of the caregiving role and determine aspects where they needed support. Caregivers valued guidance from the FECH nurse in accessing information and resources, which helped them to feel more connected to support, more prepared to care for the older person and themselves, and more secure in the caregiving role.

**Conclusions:**

Caregivers’ experiences indicated that the structured reflective FECH discussions prompted thought and provided guidance in navigating health and care systems. The FECH program appears to offer a means to address the practical, physical and psychosocial needs of informal caregivers as partners in person-centred health and social care.

**Trial registration:**

ANZCTR Trial ID: ACTRN126140011746773.

## Background

As the global population ages, increasing numbers of older people are living with chronic health problems, declining function, and frailty [1]. In Australia, an estimated 60% of people aged 65 years or older are living with at least two chronic diseases [2]. Moreover, those who turned 65 in 2015 are likely to live another 20 years, with at least 10 years destined to be with disability [3]. As older people seek to live out these years, and ultimately die, at home, family members, friends and neighbours (informal caregivers) are increasingly relied upon to provide support [4, 5]. In a recent analysis of US data, 8 million workers identified as a caregiver of an adult family member who was, most commonly, experiencing a condition related to ageing [6]. That study highlighted diversity in the informal caregiving role, with tasks ranging from transportation, providing meals and housework, to supervising medication, managing finances, and assisting with hygiene and mobilisation.

While there are undoubtedly benefits for both caregivers and care recipients from maintaining the caregiving role, the demands of caregiving can precipitate caregivers’ mental and physical health issues, also reducing employment productivity and thereby caregiver income [6, 7]. The stress of caregiving increases when a health crisis results in the care recipient’s hospitalisation, bringing additional worries for the future and greater decision-making demands [8]. Moreover, with health systems under pressure, informal caregivers take on a progressively comprehensive role supporting acutely ill patients after discharge as hospital length of stay shortens [4].

In recognition of this caregiver role, stakeholders including patients and caregivers in the US investigated characteristics of effective transitional care “to promote positive health outcomes throughout periods of acute illnesses extending from hospital to home” (p. 1120) [9]. Published evidence was integrated with experiences of stakeholders plus case studies and narratives shared by patients and caregivers to inform eight core components of transitional care, which promote positive health outcomes. Three components were directly related to including and supporting caregivers when older patients go home from hospital: *Caregiver engagement* focused on discerning caregivers’ preferences, needs and capabilities, and facilitating decision-making involving caregiver input; *Caregiver education* included preparing caregivers with skills to enable involvement in decision-making, and connecting caregivers to community resources that develop their competencies to meet the patient’s needs and their own; and *Caregiver well-being* acknowledged that acute illness is stressful for patients and those who care for them, plus the consequent need to foster coping skills and decisions that promote quality of life [9].

These findings articulate the importance of caregivers as an informed and supportive resource to help older people manage the transition from hospital to home. Moreover, they demonstrate the need for strategies to address caregivers’ concerns and build capacity to promote better health outcomes.

### The Further Enabling Care at Home program

The Further Enabling Care at Home (FECH) program was developed and trialled with family caregivers of older patients discharged from a Medical Assessment Unit (MAU) within an Australian hospital [10]. The FECH program is a telephone-based, outreach intervention designed to systematically address support needs of family caregivers of older people after hospital discharge to sustain their home-based caregiving. A detailed description of the FECH program is provided in Toye et al., 2016 [10]. In that study, the FECH program was delivered in three telephone contacts with caregivers by an experienced nurse, commencing within approximately 1 week of discharge, with the final contact occurring between 11 and 40 days (median 19 days) post-discharge. During these contacts, the FECH nurse explored the caregiver’s understanding of information provided in the patient’s copy of the discharge summary sent to the General Practitioner (GP), used a validated approach for caregivers to systematically identify and prioritise their needs, and provided guidance to access existing resources. The FECH nurse was prepared for the role with education and a previously developed resource manual providing links to information and supportive services [11].

A cornerstone of the FECH program was the caregiver-led approach using the Carer Support Needs Assessment Tool (CSNAT) [12–14]. The CSNAT was developed following literature reviews [14] and interviews with 75 bereaved caregivers; it has demonstrated satisfactory psychometric properties [12–14]. The tool has 14 items covering two domains, support for the caring role and for the caregiver’s well-being. The assessment tool is introduced to the caregiver who is given time to reflect on the questions before an assessment conversation that supports the caregiver to articulate the importance and relevance of each item. Guidance is then offered to access information and resources that address the caregiver’s prioritised needs [14].

Our team conducted a single-blind randomised controlled trial (RCT) of the FECH program with caregivers of patients aged 70+ discharged from the MAU [10]. Caregivers (*n* = 175) were randomised to either receive the FECH program and usual care (*n* = 86, intervention), or usual care alone (*n* = 89, controls). Attrition was higher in the intervention group, with completed data sets received from 62 caregivers (19.5% attrition) compared to 79 controls (8.5% attrition). The primary outcome variable was caregivers’ self-reported preparedness to care measured by the Preparedness for Caregiving Scale from the Family Care Inventory [15]. Secondary outcomes included caregiver strain measured by the Family Appraisal of Caregiving Questionnaire [16], and changes in physical and mental health measured by the SF-12v2® [17]. Results showed that family caregivers receiving the FECH program in addition to usual care were significantly better prepared to provide care and reported reduced carer strain compared to family carers who received usual care alone [10].

### Objective

In this first trial of the FECH program, we included a qualitative evaluation, via telephone interviews, to explore caregivers’ experiences and perceptions of the FECH program.

## Methods

This study component employed a qualitative descriptive design [18, 19]. This design was selected to obtain a rich description of caregivers’ first hand experiences of receiving the FECH intervention, in order to understand their perspectives of its potential value and aspects seen as more or less helpful that might inform further refinements [20, 21].

### Setting, sample and recruitment

The study setting was the 36-bed MAU within a 610-bed Australian tertiary hospital that admits over 50% of the 70,000 emergency department presentations annually, with most medical patients going to the MAU. Patients were assessed and treated on the MAU for up to 72 h before discharge or transfer to another ward. Discharge was on the premise that the patient would receive rapid follow-up from their GP or the hospital out-patient clinic. An electronically generated discharge summary was provided in hard copy to the patient with a copy forwarded to the GP.

Convenience sampling was used; caregivers who had received the FECH intervention were invited to participate in a telephone interview [21]. Following participants’ completion of the FECH program and quantitative data collection, their contact details were provided to the qualitative interviewer. The interviewer then phoned caregivers to invite participation in a telephone interview to provide feedback on their experience of the FECH program. The interviewer provided verbal information and answered participants’ questions about this qualitative component of the study, mailed a written information sheet and consent form, and scheduled the interview. Following receipt of the signed consent form, the interviewer phoned the participant at home at the appointed time to complete the interview. Participants were not contacted again.

### Data collection

Data were gathered using semi-structured interviews [19–21]. The choice of telephone, rather than face-to-face, interviews was to minimise burden on caregivers by not requiring them to accommodate a visit from the interviewer or attend an external venue, and to provide flexibility should the caregiver need or wish to reschedule. The six-question guide was structured to focus on caregivers’ experience of the FECH program [21] and is provided in Table 1. Additional probing questions were used to explore emerging ideas in more depth. The interviews were conducted by an experienced qualitative interviewer who had a nursing background. Interviews continued until data saturation was achieved, when no new information was emerging from the data [22, 23].

**Table 1:** Semi-structured interview guide providing questions and associated prompts

Question No	Question Text	Additional prompts
1	Please tell me how you have cared for your relative since he/she left hospital?	Providing for physical and/or emotional needs; speaking up for him/her; changes in caregiving role from before the admission?
2	What sorts of things help to support you to provide this care?	Information; equipment; contact with GP, practice nurse, hospital staff; community services (which ones?); social support through friends clubs, church; care plans; how accessed – organised by hospital or other, or did you have to seek out yourself
3	Is there anything that makes it harder to keep caregiving?	Physical load, isolation, fatigue; other?
4	Overall, how does being a caregiver make you feel?	
5	Please tell me about your contact with the FECH nurse?	Convenience, easy or hard to talk to; enough contact; appropriateness of telephone?
6	And how did you find the resources suggested by the FECH nurse?	Did you access these? If so, how helpful were they? And why? If not, why not?

### Data analysis

Interviews were audio-recorded and transcribed verbatim. Each transcript was checked against the recording for accuracy but, in order to minimise burden on caregivers, not returned to participants for comment. Transcripts were subjected to thematic analysis [24]. Two experienced qualitative researchers, including the interviewer, coded data by reading the transcripts line-by-line to identify salient words and sentences. The researchers met regularly to review interview transcripts looking for repetition in participants’ comments and redundancy in the data, at which point it was determined that data saturation had been reached [22, 23]. Emergent categories were grouped to generate tentative themes. NVIVO software supported axial coding [25], which confirmed final themes describing caregivers’ experiences of the FECH program.

### Trustworthiness

Strategies were undertaken to establish the trustworthiness of the findings [26, 27]. Credibility was enhanced through data collection by an experienced qualitative interviewer who understood the nursing context but was separate from the FECH program to maintain objectivity and enable participants to feel comfortable sharing both positive and negative experiences. All transcripts were checked for accuracy against audio recordings, and then read and coded independently by two qualitative researchers who came together to confirm concepts identified in the data. Direct quotes were provided to support interpretation of the data. To minimise burden on the caregivers, emergent themes were not returned to participants for verification, but rather subjected to scrutiny by the wider research team. To establish dependability, extensive meeting notes documented decisions, operational details, and team discussions as the research progressed. A description of the study setting, participants, and research processes are provided to enhance transferability to other similar contexts [26, 27].

## Findings

Twenty-one caregivers (34% response) were interviewed (median age = 67 years, range = 25-89 years, 81% female). Characteristics of the interviewed sample were compared to all caregivers who completed the FECH intervention, as shown in Table 2. There were no statistically significant differences between the sample and the overall group, although interviewed participants were more likely to be the patient’s wife and more likely to be providing social support / advocacy.

**Table 2:** Characteristics of interview participants compared to all caregivers who received the FECH program

Characteristic	Interviewed caregivers *n* = 21		All caregivers who received intervention *n* = 62		*P* value
	n	(%)	n	(%)	
Caregiver relationship to patient					0.509
Husband	1	(4.8)	4	(6.5)	
Wife	9	(42.9)	18	(29.0)	
Son	3	(14.3)	11	(17.8)	
Daughter	7	(33.3)	22	(35.5)	
Grandchild	1	(4.8)	7	(11.3)	
Caregiver age	Years		Years		0.617
Mean (SD)	64.43	(16.09)	63.13	(12.68)	
Median	67		63.50		
Range	(25–89)		(25–89)		
Caregiver highest education					0.745
Primary	1	(4.8)	3	(4.9)	
Secondary	8	(38.1)	23	(37.1)	
Trade (e.g. TAFE qualification /apprenticeship)	4	(19.1)	17	(27.4)	
Tertiary	8	(38.1)	19	(30.7)	
Duration of caring					0.928
Less than 12 months	3	(14.3)	11	(17.8)	
12–24 months	4	(19.1)	12	(19.4)	
More than 24 months	14	(66.7)	39	(62.9)	
Caregiver living with patient					1.000
No	9	(42.9)	27	(43.6)	
Yes	12	(57.1)	35	(56.5)	
Caregiver known medical problems					0.970
Yes	14	(66.7)	43	(69.4)	
No	7	(33.3)	19	(30.7)	
Types of support provided by caregiver to patient					1.000
*Multiple answers possible*					
Physical care	8	(38.1)	29	(46.8)	0.477
Emotional care	21	(100)	62	(100)	NA
Instrumental care	20	(95.2)	59	(95.1)	1.000
Social Support/Advocacy	17	(81.0)	39	(63.0)	0.068
Caregiver frequency of contact with patient by phone or in person					0.865
At least daily	18	(85.7)	51	(82.3)	
At least weekly	3	(14.3)	9	(14.5)	

Interviews ranged from nine to 42 min in duration. Qualitative analyses generated three major themes that described family caregivers’ perceptions of: The experience of caregiving; The experience of receiving FECH program support, which described how caregivers felt more connected to support, more prepared to care, and more secure in the caregiving role; and Caregivers’ suggestions for improvement.

### Theme 1: the experience of caregiving

Family caregivers’ experiences of caregiving after the older person’s hospitalisation were woven through the interviews. Caregivers described a busy and complex role that most commonly included checking on the older person, and providing meals, medications, transport, cleaning, help with hygiene, and advocacy*.* Some caregivers found that hospital admission had benefitted the older person, potentially easing the caregiving role such as when, *“he got more rehab [ilitation] so he’s been a bit more mobile” (C113).* However, others noticed increased demands on them as the effects of illness and hospitalisation left the older person more vulnerable. For one patient’s wife, her husband’s return home from hospital seemed life-changing because:

My husband is not the man he used to be . . . I have to think for him . . . know all his needs that he has because he can’t tell me . . . . I come back home and he was [sic] fully dependent on me. (C25)

In the face of this perceived vulnerability, caregivers expressed a need to stay physically close to reassure the older person when, “*there is quite a definite change*. *.*. *he now gets frightened if he can’t see me” (C53);* or themselves such as, *“when he had a shower, he liked to be independent*. *.*. *but I wasn’t sure enough and I always stayed in the next room” (C163). C*aregivers who lived separately from the older person also sought this closeness through, *“daily contact*. *.*. *emotional as well as practical” (C23)*.

Providing care for a family member could be a positive and fulfilling experience. One caregiver had found that, *“it gives me satisfaction. It makes me feel important or worthwhile” (C63),* while an adult son reported how, *“caring for Dad has had no negative impacts on me, I actually enjoy spending time with him” (C127).* However, the sense of satisfaction could be tinged with ambivalence. For example, one patient’s wife found that caregiving, *“makes me feel that I am doing something worthwhile, that I have managed to stand up and be what [Name] needs me to be” (C53),* yet revealed, *“because I love my husband I’m more than willing. Although I’m often not willing, does that make any sense?” (C53).*

The increased demands of caregiving after hospital discharge could leave caregivers feeling depleted, with implications for their own health. As one spouse caregiver remarked, *“I’m getting older myself, I’m breaking down and that’s where the hard part comes” (C25).* Adult children could find it tiring to juggle multiple responsibilities, *“I’ve got fatigue but I think it’s more related to work. Right yes, I’m actually going to the doctor actually myself today” (C57).* Along with the physical load, there was the emotional impact of watching a loved one’s decline. For one patient’s wife it was, *“sad to see him like he is” (C69).* Uncertainty about the older person’s medical condition added to the anguish and a daughter described, *“that wave of uneasiness*. *.*. *because we could not control it. And we didn’t know what we were trying to [help mum] deal with” (C158).*

Significant support was noted as being available after discharge through the GP, local government, caregiver support associations, and private providers. However, some caregivers found it difficult to access the seemingly complex health and care system. A patient’s wife recalled, *“I was a little overwhelmed with all the different areas that I needed to contact and wasn’t quite sure how to go about it” (C53).* Another described the daunting experience of interacting with service providers:

. . . its long, it’s tedious and it’s complicated, and particularly for people that are in their 80s the paperwork is enormous . . . baring your feelings as well is enormous, particularly if you don’t like asking for assistance. And sometimes you can get quite emotional . . . it’s the system you just don’t feel like navigating. (C29)

It was amidst these experiences of uncertainty, fatigue and, for some, feeling overwhelmed that caregivers were offered the FECH program through the three telephone calls from the FECH nurse, integrating the CSNAT approach and links to support.

### Theme 2: the experience of receiving FECH program support

Most caregivers found their interactions with the FECH nurse to be positive experiences. One patient’s wife said simply, *“I felt really good after I had spoken to her [FECH nurse]” (C69).* Caregivers considered the telephone a convenient mode of contact considering the multiple demands on their time:

With so many medical appointments and things, I appreciated it being over the phone . . . easier in a way, just sitting and talking, rather than to go somewhere for an interview or have someone else come over. (C63)

Flexibility was key, and caregivers appreciated that when, “*something of mine suddenly came up and it was easy*. *.*. *[to] reorganise” (C116).*

The FECH discussions were thought sufficiently structured to canvas caregivers’ needs, being, *“fairly comprehensive*. *.*. *covered all areas I think that certainly might apply to my situation” (C116),* yet responsive to their priorities, *“definitely reflected on what I had particularly said there, like in terms of me and what I wanted more information about” (C140).* For some, conversations with the FECH nurse presented a rare opportunity to reflect upon their caregiving role and explore possibilities. These reflective discussions tended to make the caregiving role more visible and, *“highlighted it and*. *.*. *acknowledged all the stuff that we actually do” (C135).* In describing these interactions with the FECH nurse, caregivers described experiences of feeling more connected to support, feeling more prepared to care for the older person and themselves, and, consequently, feeling more secure in the caregiving role.

#### Feeling more connected to support

In these interviews, caregivers spoke of the comfort they derived from sharing their concerns with the FECH nurse. One caregiver remarked, *“it’s just great to talk to somebody. Yeah, nobody else wanted to talk about it” (C29).* These data suggested that caregivers saw the FECH nurse as independent, knowledgeable, and focused on their needs. Consequently, they could feel comfortable sharing their feelings:

That’s how I felt, that I had somebody who was phoning me up going “How are you doing?” And [me] . . . out of earshot of mum [saying], “it’s frightening” and being able to explain that . . . [to] voice that. And say “Well we are kind of scared for mum at the moment, but we don’t want to show it to her all the time. And what’s going on?” (C158)

Beyond a connection with the FECH nurse, caregivers were also helped to connect with community-based health and care providers. They valued guidance as to where to access appropriate support, plus assistance to connect with that support if required:

She pointed me in the right direction . . . in fact she did contact a couple of people for me . . . I was a little overwhelmed . . . wasn’t quite sure how to go about it . . . and that was very helpful. (C53)

In the midst of uncertainty about the older person and the future, caregivers also valued having someone to contact should the unexpected occur:

It does make you feel better when you know that there is something out there . . . you don’t feel so sort of isolated . . . if it does come up you just know that you can do something more, by just simply lifting up the phone. It’s ideal. (C96)

The implication was that, with timely access to information and supportive resources, caregivers felt more prepared to care for the older person and sustain themselves in the caregiving role.

#### Feeling more prepared to care

It was evident that caregivers appreciated the FECH nurse’s guidance, which was informed by nursing expertise and an understanding of the local health care context. One patient’s wife valued advice about how she might advocate more productively for her husband in an upcoming GP appointment:

I was going to approach the doctor . . . [about] something I wasn’t happy about . . . I was going to go in there “with my boots on” . . . talking it over with [FECH nurse], it was wonderful because she was able to give me . . . a better side of looking at it, which was much better for me in the long run. (C96)

Another adult daughter showed how the FECH nurse guided her to think about the longer term and how to navigate what was likely to be ahead:

Nobody wants to, to look into that future crystal ball . . . you haven’t got 30 – 40 years more with them . . . she was really, really, she was honest about things. And she kept it real but she was also really supportive of me. (C158)

With a sense that knowledge is power, caregivers reported feeling more able to take action for themselves, *“I’ve always been a little unsure as to what help I could get and what my options were*. *.*. *I’m more confident to ask now” (C53).* It was perhaps for this reason that caregivers saw that receiving the FECH program soon after discharge would be most helpful because, *“at the beginning, the quicker you can get access to information the better it is” (C96).* Armed with information, caregivers tended to feel more in control as evidenced by:

. . . before we started all this we had never experienced being rushed to hospital . . . now I have . . . numbers that I can ring if I need help . . . a bit like a girl guide, I’m prepared now. (C145)

It was also evident that as they acknowledged the demands of their role and understood more about the older person’s likely care needs, caregivers started to think about what they needed to sustain themselves in that role. As this participant explained, *“You just get caught up in it and don’t realise “oh gees I am really tired” . .*. *it’s good to have someone “check in” and it makes you check in on yourself” (C113*). In doing so, some felt afforded permission to do things for themselves that came from *“self-awareness of [sic] if you do not look after yourself, how can you look after and care for other people?” (C158).*

#### Feeling more secure in the caregiving role

These data suggested that the connection to support offered by the FECH nurse and increased confidence contributed to a sense of empowerment. As one adult daughter stated,, *“I’ve got [FECH nurse’s] phone number*. *.*. *that’s my safety net” (C128*); while a patient’s wife remarked, *“elderly people*. *.*. *normally we are sort of second [class] citizens so to speak. But there was back-up there and that made you a lot stronger in the role” (C96).*

### Theme 3. Caregivers’ suggestions for improvement

As described earlier, flexibility was key and caregivers found it helpful that the telephone calls could be organised, and reorganised, to suit their schedules. However some also saw the need for more flexibility in the content of the call. Considering the physical and emotional demands on caregivers, some found that, *“by the end of that hour and a half I was pretty wrung out” (C128).* A suggestion was to integrate other forms of communication such as:

She spent most of her time giving me lists of addresses . . . she could have spent less time and . . . posted the thing out . . . I spent all of my time writing down instead of trying to think. (C165)

Caregivers’ time was precious and while they found that connection to the FECH nurse helped them to feel prepared and secure, it was important that these contacts were as productive as possible. Nevertheless, one caregiver surmised, *“It wasn’t as hard as it would have been without [the FECH nurse]” (C53).*

## Discussion

These interviews described a complex landscape of caregiving for an older person following the person’s hospital discharge, the physical and emotional loads borne by caregivers, and difficulty of navigating service provision. Findings of the current study support the notion that patients and caregivers are assuming the “burden of treatment” (p. 281) as care shifts from hospitals to the community [4]. In this context, the capacity of patients and those who support them to manage this burden is seen to be contingent upon their social skills (ability to elicit cooperation of others) and social capital (ability to access information and resources). Interventions that strengthen supportive networks around patients, equip them to navigate the system, and increase social capital are likely to increase effective use of healthcare [4]. Moreover, interventions that monitor the load on patients and caregivers, and its effects, can build capability to perform required healthcare and support tasks [4].

In the current study, the telephone-based FECH program put caregivers in post-discharge contact with a registered nurse with knowledge of the hospital and community settings, who used a systematic caregiver-led conversation to identify, prioritise and address their support needs. From the caregiver perspective this telephone-based program was timely, delivered away from the busy hospital environment but at a time when demands on caregivers were significant. Participants reported experiences of the program, and indicated how this connection to the FECH nurse helped them to feel more prepared to care and more secure in the caregiving role. There were three key elements of the FECH program that caregivers indicated offered particular value and meant that the program was especially appropriate from their perspectives.

First, the structured caregiver-led CSNAT conversation prompted thought and helped make the caregiving role more visible. For some caregivers, this prompted them to think about their own self-care. Similar findings were obtained when the CSNAT approach was implemented with caregivers of cancer patients in community palliative care [28] and in the context of motor neurone disease (MND) care [29] and dementia community care [30]. In those studies, family caregivers reported that the focus on their well-being was both validating and reassuring, particularly as they worked together with the health practitioner to address their prioritised needs [28, 29]. Moreover, caregivers reported feeling listened to and being more aware of the scope of their role, understanding that the patient suffers if the caregiver isn’t coping. In the current study, caregivers described the increasing demands of supporting an older family member whose health is deteriorating. For some, the reflective discussions with the FECH nurse served to highlight the complexity of the role that caregiver themselves may not have recognised. Receiving the FECH program, with the CSNAT approach at its core, during the “window of opportunity” offered by hospital discharge could put tangible support in place when caregiving demands were at a peak.

Second, caregivers valued the personal connection (albeit by telephone) with the FECH nurse, which tended to reduce isolation and engendered the sense of a ‘safety net’, and guidance to access supportive resources outside of the hospital. Substantial evidence suggests that linking caregivers to information and resources for use following a patient’s discharge from hospital may reduce hospital readmissions. As evidence, a metaanalysis of interventions involving older patients’ caregivers in discharge planning included 11 studies, with just over half the interventions delivered by nurses [31]. Six studies reported a 25% reduced risk of 90-day readmission from integrating caregivers into discharge planning (*n* = 1773). Similarly, the pooled intervention effect in five studies reporting 180-day readmissions (*n* = 2037) indicated a 24% reduced risk of readmission. While these interventions differed from the caregiver-focused FECH program, which is delivered post-discharge, all included some component that linked the caregiver to community resources [31].

Third, findings from the current study indicate that the FECH program increased caregivers’ confidence in their ability to navigate the systems of health and community care. It was evident that the FECH nurse’s experience, training and familiarity with the healthcare context were critical to the factors identified by caregivers as beneficial. As well as the sense of security in feeling a connection to the FECH nurse, they felt more able to use productive approaches in their interactions with healthcare providers. In other studies mentioned earlier in this paper, the reflective CSNAT conversation has been repeated at intervals enabling caregivers to monitor their own needs and maintain links with service providers who can offer further guidance to address areas of increasing demand [28–30]. There is potential for the FECH program to be adapted in this way, on the basis of individuals’ needs, perhaps leading to an ability to undertake the reflective process without nurse prompting and access further guidance from established caregiver support organisations.

Limitations include the possibility that caregivers who preferred not to receive further phone calls declined or didn’t respond to the invitation to participate. Therefore, the perspectives of caregivers who found the FECH contacts burdensome or those experiencing particularly high levels of stress may not be adequately represented in the findings. Additionally, this study was conducted in one setting in the Australian context in which rapid patient discharge was the norm. The findings may be transferable, however, to other similar settings in countries where similar healthcare services are available. For example, in the US, a number of states have passed the Caregiver Advise, Record, Enable (CARE Act) legislation requiring that hospitals identify family caregivers who are informed about plans for the patient’s discharge and educated to provide follow-up care [32]. The FECH program might be a useful addition to such education.

Future work to evaluate the program in other settings and over a longer time frame is also appropriate and adaptations of the program merit consideration. For example, an approach in which the timing of FECH program and mode of information delivery are responsive to caregivers’ needs and preferences may aid in reducing the attrition rate observed in the intervention group in the current study (RCT component) of 19.5%.

## Conclusions

As hospital length of stay shortens, and family caregivers of older people are increasingly relied upon to provide complex post discharge support, the FECH program appears to offer a means to address practical and psychosocial needs of family caregivers, who are “crucial partners” (p. 423) in person-centred health and social care [33]. These caregivers indicated that the FECH program recognised and supported their contributions with links to relevant information and resources. Receiving this support in the first month after an older patient’s hospital discharge was valuable and tended to build caregivers’ confidence and a sense of security. Provision of the FECH program over a longer time frame involving repeated implementation of the CSNAT approach offers potential to empower caregivers with problem-solving skills and increased social capital (information and resources) to more effectively navigate the healthcare system [4]. The systematic approach embodied by the FECH program is consistent with sustainable healthcare in that the caregiver’s voice is heard and strategies build capacity for home-based caregiving. Future work to lengthen and further tailor delivery to caregivers’ preferences is warranted.

## Abbreviations

CARE: Act Caregiver Advise, Record, Enable legislation
CSNAT: Carer Support Needs Assessment Tool
ED: Emergency Department
FECH: Further Enabling Care at Home program
GP: General Practitioner
MAU: Medical Assessment Unit
MND: Motor Neurone Disease
RCT: Randomised Control Trial
US: United States of America
